# Genome-wide association study and biological pathway analysis of the *Eimeria maxima* response in broilers

**DOI:** 10.1186/s12711-015-0170-0

**Published:** 2015-11-25

**Authors:** Edin Hamzić, Bart Buitenhuis, Frédéric Hérault, Rachel Hawken, Mitchel S. Abrahamsen, Bertrand Servin, Jean-Michel Elsen, Marie-Hélène Pinard - van der Laan, Bertrand Bed’Hom

**Affiliations:** UMR1313 Animal Genetics and Integrative Biology Unit, AgroParisTech, 16 rue Claude Bernard, 75005 Paris, France; UMR1313 Animal Genetics and Integrative Biology Unit, INRA, Domaine de Vilvert, 78350 Jouy-en-Josas, France; Department of Molecular Biology and Genetics, Center for Quantitative Genetics and Genomics, Aarhus University, Blichers Allé 20, P.O. Box 50, 8830 Tjele, Denmark; UMR1348 Physiology, Environment and Genetics for the Animal and Livestock Systems Unit, INRA, Domaine de la Prise, 35590 Saint Gilles, France; Cobb-Vantress Inc., Siloam Springs, AR 72761 USA; UMR1388 Genetics, Physiology and Breeding Systems, INRA, 24 chemin de Borde-Rouge, 31326 Castanet-Tolosan, France

## Abstract

**Background:**

Coccidiosis is the most common and costly disease in the poultry industry and is caused by protozoans of the *Eimeria* genus. The current control of coccidiosis, based on the use of anticoccidial drugs and vaccination, faces serious obstacles such as drug resistance and the high costs for the development of efficient vaccines, respectively. Therefore, the current control programs must be expanded with complementary approaches such as the use of genetics to improve the host response to *Eimeria* infections. Recently, we have performed a large-scale challenge study on Cobb500 broilers using *E. maxima* for which we investigated variability among animals in response to the challenge. As a follow-up to this challenge study, we performed a genome-wide association study (GWAS) to identify genomic regions underlying variability of the measured traits in the response to *Eimeria maxima* in broilers. Furthermore, we conducted a post-GWAS functional analysis to increase our biological understanding of the underlying response to *Eimeria maxima* challenge.

**Results:**

In total, we identified 22 single nucleotide polymorphisms (SNPs) with q value <0.1 distributed across five chromosomes. The highly significant SNPs were associated with body weight gain (three SNPs on GGA5, one SNP on GGA1 and one SNP on GGA3), plasma coloration measured as optical density at wavelengths in the range 465–510 nm (10 SNPs and all on GGA10) and the percentage of β2-globulin in blood plasma (15 SNPs on GGA1 and one SNP on GGA2). Biological pathways related to metabolic processes, cell proliferation, and primary innate immune processes were among the most frequent significantly enriched biological pathways. Furthermore, the network-based analysis produced two networks of high confidence, with one centered on large tumor suppressor kinase 1 (LATS1) and 2 (LATS2) and the second involving the myosin heavy chain 6 (MYH6).

**Conclusions:**

We identified several strong candidate genes and genomic regions associated with traits measured in response to *Eimeria maxima* in broilers. Furthermore, the post-GWAS functional analysis indicates that biological pathways and networks involved in tissue proliferation and repair along with the primary innate immune response may play the most important role during the early stage of *Eimeria maxima* infection in broilers.

**Electronic supplementary material:**

The online version of this article (doi:10.1186/s12711-015-0170-0) contains supplementary material, which is available to authorized users.

## Background

Coccidiosis is an animal parasitic disease caused by protozoans belonging to the Coccidia subclass. In the chicken, seven species of the genus *Eimeria* are infectious and cause coccidiosis: *E. brunetti*, *E. necatrix*, *E. tenella*, *E. acervulina*, *E. maxima*, *E. mitis*, and *E. praecox*. *E. maxima* is the most immunogenic of the seven species [1] and mostly infects the lining of the jejunum, causing mucoid enteritis [2]. Chicken coccidiosis is one of the most common and costly diseases currently affecting the poultry industry, with worldwide costs caused by production losses as well as by prevention and treatment actions that are estimated to exceed USD 3 billion per year [3, 4]. Current coccidiosis management is based on the use of anticoccidial drugs and vaccination [5]. The first anticoccidial drugs, sulfonamides, began to be used in the early 1940s, and then over the years, several different classes of drugs were developed and extensively used for the control of coccidiosis in broiler production [6]. However, the future use of anticoccidial drugs has caused concern due to the tendency of *Eimeria* species to rapidly develop resistance to drugs [7] as well as public dissatisfaction regarding the presence of chemical residues in food. In addition, the development of efficient multiple-species live vaccines is primarily limited by their high economic costs [5].

Due to these issues, the current control programs must be expanded using a complementary approach, including the application of genetics to improve the host response to *Eimeria* infection. Genetic approaches and manipulations have been shown to have a small effect at each generation, but also a cumulative effect and is a long-term, cost-effective and environmentally friendly way of keeping livestock animals healthy [8]. The first studies indicating that genetic diversity may account for differences in susceptibility to coccidiosis were published in the 1940s and 1950s [9, 10]. Considerable variation in coccidiosis susceptibility has been observed between different chicken breeds [11]. The first successful performance of divergent selection for resistance and susceptibility to acute cecal coccidiosis was performed by Johnson and Edgar [12]. Likewise, marked differences in the response to *Eimeria* infection were observed between inbred and outbred lines [13, 14]. The availability of genome-wide dense markers has enabled the identification of several quantitative trait loci (QTL) regions associated with resistance to *Eimeria tenella* and *Eimeria maxima* in experimental populations [15–17]. In addition, several genes that are located in highly significant previously detected QTL [16] were also found to be differentially expressed in a follow-up transcriptome study [18].

Recently, we conducted a large-scale challenge study with Cobb500 broilers using *E. maxima* as the infective agent, and high variability was observed in the measured traits among challenged animals [19]. The measured traits included a wide range of physiological, immunological and disease resistance parameters. This study is a follow-up on the aforementioned large-scale challenge study in which we assessed the effects of the *E. maxima* challenge on the measured traits and also evaluated the level of variability of the measured traits. Taking advantage of the size and structure of this large-scale challenge study, we performed a genome-wide association study (GWAS) to identify genomic regions underlying the broiler response to *E. maxima*. In addition, based on the results of the GWAS, a post-GWAS functional analysis was performed to further understand the biology of the underlying response to the *E. maxima* challenge. The functional analysis comprised the following two independent approaches: a biological pathway analysis based on the publicly available Kyoto encyclopedia of genes and genomes (KEGG) pathways and a network-based analysis that was performed using the ingenuity pathway analysis (IPA) software.

## Methods

### Ethics statements

All procedures were conducted under License No. A176661 from the Veterinary Services, Charente Maritime, France and in accordance with guidelines for the Care and Use of Animals in Agricultural Research and Teaching (French Agricultural Agency and Scientific Research Agency) (http://www.gouvernement.fr/en/culture-education-and-research).

### Experimental population and phenotyping

**Fig. 1:**
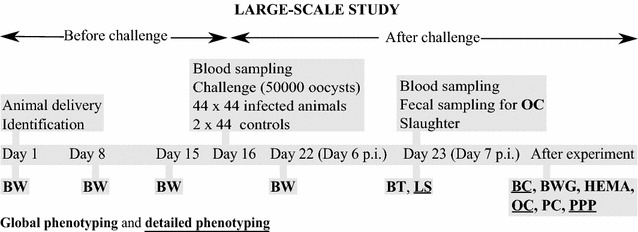
Experimental layout of the large-scale challenge study. In total, 2024 1-day-old broilers were used in the experiment with 88 control animals and 1936 challenged animals. The challenge was performed on day 16 of the experiment by inoculating 50,000 *Eimeria maxima* oocysts. Traits were measured at two levels: “global phenotyping” and “detailed phenotyping”. Global phenotyping was performed on all animals and included body weight (BW), plasma coloration (PC), body temperature (BT) and hematocrit (HEMA) levels. Detailed phenotyping was performed on the subset of 176 animals and included lesion score (LS), oocyst count (OC), plasma protein profiles (PPP), and blood composition (BC). Body weight gain (BWG) was calculated using the following formula (BW at day 22-BW at day 15)/BW at day 15. PC is optical density of blood plasma measured for 44 wavelengths (every 5 nm, 380–600 nm) on days 15 and 22. BT was measured at day 23, and HEMA was measured from blood samples obtained on days 16 and 23. BC, PPP, OC and LS were assessed using samples obtained on day 23. Please refer to Hamzic et al. [19] for a detailed description of the trait measurements

In this study, we used phenotype data collected during a large-scale *Eimeria maxima* challenge study on Cobb500 broilers [19]. The challenge study was performed using 2024 Cobb500 broilers randomly distributed in 44 (challenge) and two (control) litter pens (3 m × 1 m) each containing 44 birds. For the GWAS, we used only data collected from the challenged animals, with control animals excluded from the analysis. Traits were measured at two levels: “global phenotyping”, which was performed on all animals and “detailed phenotyping”, which was performed on a subset of 176 animals. The experimental layout of the challenge is presented in Fig. 1.

BWG, HEMA, BT and PC were measured on all animals. BWG was calculated as BWG = (BW at day 22-BW at day 15)/BW at day 15. We used relative BWG with respect to day 15 to focus solely on the BWG in response to the challenge. HEMA levels and PC were measured from the blood samples that were collected on days 16 and 23.

The subset of 176 animals was chosen by selecting two birds among those with the lowest and two birds among those with the largest BWG from each challenged animal pen. Animal selection within each pen was based on the ranking per pen. The subset of 176 animals with extreme BWG values was used for further detailed phenotyping. Detailed phenotyping included LS (duodenum and jejunum), OC, BC and PPP. PPP was assessed using capillary electrophoresis, which separates the protein components into five major fractions by size and electrical charge: prealbumins, albumins, α-1 globulins, α-2 globulins, α-3 globulins, β-1 globulins, β-2 globulins and γ globulins. BC included blood cell count (BCC) and red blood indices (RBI). A detailed description of the methodologies used for measuring all traits was reported by Hamzic et al. [19].

### Genotype data

DNA was extracted from blood samples obtained from 1972 animal samples at day 16 of the experiment. Genotyping was performed using a 580 K Affymetrix^®^ Axiom^®^ HD genotyping array (Affymetrix, Santa Clara, USA) [20] at a commercial laboratory (GeneSeek, Lincoln, USA). Quality control of the genotype data was performed using PLINK 1.9 [21–23] and included the sample call rate (>98 %), SNP call rate (>98 %), minor allele frequency (>2 %) and removing SNPs with extreme F-statistic values. A total of 138,568 SNPs out of 580,961 were removed during the quality control (See Additional file 1: Table S1). SNP thresholds and sample call rates were set at 98 % and all SNPs and individuals with a call rate less than 98 % were excluded from the analysis. We did not perform imputation of the residual missing genotypes. The F-statistics distribution for the SNPs that remained after the quality control steps was assessed, and all SNPs outside the adjusted interquartile range were excluded from the analysis. The interquartile range for skewed distribution of F-statistics was calculated as suggested by Hubert and Vandervieren [24]. Sex was assessed using PLINK 1.9 by analyzing SNPs on the Z and W chromosomes. Detected females were included in the analysis with sex considered as a covariate (See Additional file 1: Table S1). Finally, after quality control, we obtained a dataset that included 443,587 SNPs distributed along 28 autosomal chromosomes, two linkage groups (LGE22C19W28_E50C23 and LGE64) and chromosome Z. Mean, median and standard deviation for base pair distances between neighboring SNPs across chromosomes are in Table S2 (See Additional file 2: Table S2).

### Genome-wide association analysis

The genome-wide association analyses were performed using the Genome-wide Efficient Mixed Model Association (GEMMA) algorithm [25] for the univariate linear mixed model (LMM). In this study, we used Cobb500 broilers, which are the final products of a four-way crossbreeding scheme, indicating the presence of a strong population structure. The linear mixed model approach was used since this method has been proven to successfully account for population structure in association mapping studies [26]. In the context of the LMM, the correction for population structure is performed by creating the genomic relationship matrix (**K**) that models the structure present in the analyzed population by estimating the contribution of genetic relatedness to the phenotypic variance. **K** presents a pairwise relationship between individuals, and its structure is also influenced by population structure, family structure and cryptic relatedness.

The model used for the analysis is presented in expression (1):

1 $${\mathbf{y}} = {\mathbf{W}}\varvec{\alpha}+ {\mathbf{x}}\upbeta + {\mathbf{u}} + {\varvec{\epsilon}},$$

where **y** is an n-vector of observations (or trait measurements) for n individuals, **W** is an n × c matrix of covariates which contains information about pen and sex including a column of 1s for the general mean; **α** is a c-vector of the corresponding coefficients, including the intercept, **x** is an n-vector of marker genotypes, β is the effect size of the marker, **u** is an n-vector of polygenic effects, and **ε** is an n-vector of residual effects.

For polygenic effects, **u** follows a multivariate normal distribution (MVN_n_) $({\text{u}}\sim {\text{MVN}}_{\text{n}} \,(0, \lambda \tau^{ - 1} {\mathbf{K}}))$, with λ indicating the ratio between the genetic variance (more precisely the variance explained by the SNPs) and variance of the residuals, τ^−1^ is the variance of the residuals, and **K** is a known n × n, which is the genomic relationship matrix, calculated using a n × p matrix of genotypes (**X**). Expression (2) was used to calculate **K** where *x*_*i*_ is the *ith* column representing genotypes of the *ith* SNP in the **X** matrix, $\overline{{x_{i} }}$ is the sample mean, and **1**_***n***_ is an n × 1 vector of 1s. Residual effects are presented as an **ε** vector of length n following the multivariate normal distribution (MVN_n_) $(\varepsilon \sim {\text{MVN}}_{\text{n}} \,(0,\tau^{ - 1} {\mathbf{I}}_{n} ))$ where **I**_n_ is an n × n identity matrix.

2 $${\mathbf{K}} = \frac{1}{p}\mathop \sum \limits_{t = 1}^{p} \left( {x_{i} - 1_{n} \overline{{x_{i} }} } \right)\left( {x_{i} - 1_{n} \overline{{x_{i} }} } \right)^{T} .$$

To correct for multiple hypothesis testing, the false-discovery rate (FDR) was calculated for each SNP from the distribution of p-values: SNPs with an FDR less than 0.1 were considered significant [27]. The results are shown in Manhattan plots constructed by the qqman R package [28].

### Biological pathway analysis

In general, biological pathway analysis is used to test the association between a curated set of genes (biological pathways) and a trait of interest. This approach tests for the cumulative effect across many genes, which enables the detection of effects at the biological pathway level. Biological pathway analysis has been conducted as described in the following paragraphs. This analysis was divided into two main steps: (1) assigning SNPs to their corresponding annotated genes and assigning these genes to their corresponding biological pathways (KEGG), and (2) statistical testing for the biological pathway (set of genes with assigned list of SNP) based on the test statistics obtained in the genome-wide association analysis.

### Assigning SNPs to genes and biological pathways

The SNPs used in the GWAS were mapped to the ICGSC Gallus_gallus-4.0 chicken genome assembly (GCA_000002315.2) using the NCBI2R R package [29]. A list of annotated genes corresponding to the SNPs used in the genome-wide analysis was retrieved. For this study, the publicly available biological KEGG pathways were downloaded using the Bioconductor KEGGREST package [30], and the curated genes were assigned to specific pathways. The biological KEGG pathways represent a collection of biological pathway maps that integrate many units, including genes, proteins, RNAs, chemical compounds, and chemical reactions, as well as disease genes and drug targets, which are stored as individual entries in other KEGG databases. The chicken genome KEGG PATHWAY contains 162 pathways associated with 4342 genes. The list of genes with assigned SNPs from the GWAS and the list of genes with assigned biological pathways (KEGG) were combined, ultimately resulting in a list of 52,204 SNPs assigned to 162 biological KEGG pathways (See Additional file 3: Table S3).

### Statistical analysis for biological pathway analysis

As described in the previous step, a set of SNPs that were mapped to genes were further assigned to the corresponding biological pathways. For each biological pathway that was characterized by a set of SNPs, an appropriate summary of statistics was constructed as described in detail by Jensen et al. [31]. The statistics summary was based on the negative log-transformed p values from the association of individual SNPs to the traits. By summing these negative log-transformed p values, we imitated a genetic model that captures variants with small to moderate effects [32, 33].

The observed summary statistics for a particular set of SNPs were compared with an empirical distribution for the summary statistics of random samples of SNP sets of the same size using a permutation approach. Considering that the distribution of summary statistics is affected by a correlation structure of closely linked SNPs, as the consequence of linkage disequilibrium, the following procedure was used for statistical testing.

The vector of observed SNPs with corresponding test statistics was ordered according to the physical position on the genome. SNPs were then mapped to genes and consequently to a biological pathway as described in the first step. The elements in this vector were numbered 1,2,…,N, and the permutation was performed in two steps. The first step included randomly picking an element (e_j_) from this vector. This jth test statistic was the first element in the permuted vector, and the remaining elements were ordered e_j+1_, e_j+2_, …, e_N_, e_1_, e_2_, …, e_j-1_ according to their original numbering. Therefore, all elements from the original vector were then shifted to a new position starting with e_j_; however, the gene position was kept fixed with respect to the original one. The second step involved the computation of summary statistics for each set of SNPs based on the original position of the set of SNPs in the original vector of test statistics. The connections between SNPs and genes were broken while keeping the correlation structure among the test statistics. Steps 1 and 2 were repeated 1000 times, and from this empirical distribution of summary test statistics for each set of SNPs, a *p* value was obtained. This empirical p value corresponds to a one-sided test of the proportion of randomly sampled summary statistics that were larger than the observed summary statistic with the arbitrary significance level set to 0.01.

### Network-based analysis

The network modeling was performed using the ingenuity pathway analysis (IPA) tool as a complementary approach to the KEGG biological pathway analysis. The ingenuity pathways database is the manually curated database of previously published relationships on human and mouse biology [34]. The gene input list included genes that contained SNPs with p values below the inferred genome-wide threshold (P < 10^−4^) for all traits on which GWAS was performed (See Additional file 4: Table S4). In this manner, we restricted the list of putative candidate genes and exploited their documented interactions in biological pathways related to the study. The lists of SNPs (P < 10^−4^) were assigned to their respective genes using the biomaRt R package [35], producing the gene list for each trait used in the GWAS. The obtained Human Genome Gene Nomenclature Committee (HGNC) identifiers were mapped onto networks that are available in the Ingenuity Pathway repository. In the case of PC, we merged all gene lists obtained for individual wavelengths into one collective gene list. Furthermore, we also created a global list of genes by combining all gene lists together. In summary, we performed IPA using 27 gene lists of which 25 gene lists corresponded to each individual trait, a gene list for PC obtained by combining gene lists for all wavelengths and the global list of genes.

## Results

### Experimental population and phenotyping

In this study, we analyzed the data collected during the large-scale challenge study on Cobb500 broilers with *E. maxima* as the infective agent [19]. The experimental scheme of the large-scale challenge study is illustrated in Fig. 1. The traits were measured at two levels: “global phenotyping” performed on all 1936 challenged animals and “detailed phenotyping” performed on a subset of 176 animals (Fig. 1). To form the subset of 176 animals, two animals among those with the lowest and two animals among those with the highest body weight gain (BWG) were selected from each pen containing challenged animals.

The traits measured on all challenged animals included BWG, plasma coloration (PC) measured as optical density (OD) of blood plasma in the 380–600 nm range, body temperature (BT) and hematocrit level (HEMA). In addition to these traits, animals from the subset of 176 were phenotyped for lesion score (LS), oocyst count (OC), blood composition (BC) and plasma protein profiles (PPP) (See Additional file 5: Table S5). BC included two sets of measurements: blood cell count (BCC) and red blood indices (RBI). BCC included erythrocyte, leukocyte, lymphocyte, heterophil, and thrombocyte counts as well as the percentage of lymphocytes and heterophils of the total number of leukocytes. RBI included hemoglobin content, mean corpuscular volume (MCV), mean corpuscular hemoglobin (MCH) and mean corpuscular hemoglobin concentration (MCHC). PPP were assessed using protein capillary zone electrophoresis, and the profiles included the following fractions: prealbumin, albumin, α1-globulin, α2-globulin, α3-globulin, β1-globulin, β2-globulin, and γ-globulin. Detailed results from the large-scale challenge study were reported by Hamzic et al. [19]. Descriptive statistics of the traits measured on the challenged animals are in Table S5 (See Additional file 5: Table S5).

### Genome-wide association study (GWAS)

In total, 22 SNPs were significantly associated (q value <0.1) with the measured traits and were distributed over five chicken chromosomes (See Additional file 6: Table S6). The most significant SNPs were associated with BWG, PC for wavelengths from 465 to 510 nm and the percentage of β2-globulin in blood plasma of one of the PPP fractions.

**Fig. 2:**
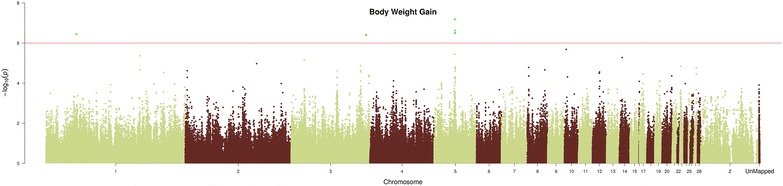
Manhattan plot for body weight gain. Manhattan plot of genome-wide −log10 (p values) for body weight gain. P values were adjusted using the false-discovery rate (FDR) at a significance level of q value <0.1. SNPs labeled in *green* have q value <0.1

The GWAS identified five SNPs that were significantly associated with BWG (Fig. 2), and the quantile–quantile (Q–Q) plot for BWG showed a large deviation from the distribution under the null hypothesis, indicating that strong associations were observed (See Additional file 7: Figure S1). The five observed SNPs are located on GGA1, 3 and 5. These SNPs explain 7.5 % of the total variance for BWG between days 15 and 23 of the challenge study. The significantly associated genomic region on GGA5 was located in the upstream region of the *THBS1* gene, which encodes the multi-domain matrix glycoprotein termed thrombospondin-1. Similarly, the significantly associated genomic regions on GGA1 and GGA3 are in the vicinity of *MGAT4C* and *KCNK3*, respectively.

**Fig. 3:**
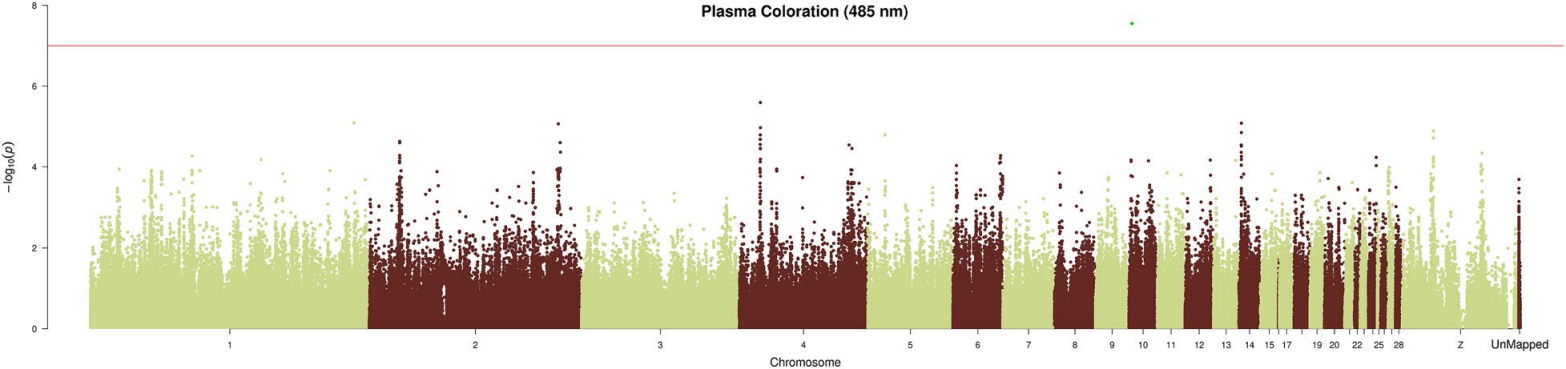
Manhattan plot for plasma coloration (485 nm). Manhattan plot of genome-wide −log10 (p values) for plasma coloration (485 nm). P values were adjusted using the false-discovery rate (FDR) at a significance level of q value <0.1. SNPs labeled in *green* have q value <0.1

For PC, the AX-75604378 SNP located on GGA10 was significantly associated with PC values measured for wavelengths ranging from 465 to 510 nm (Fig. 3). Figure 3 shows the Manhattan plot and Q–Q plot for PC measured at 485 nm. The Q–Q plot shows a strong deviation from the distribution under the null hypothesis, which indicates the presence of a strong association between the SNP and PC values (See Additional file 8: Figure S2). The associated SNP explains between 2.1 and 2.3 % of the total variance depending on the measured wavelength (See Additional file 6: Table S6). The AX-75604378 SNP is a non-synonymous polymorphism present in the *MAN2C1* gene.

**Fig. 4:**
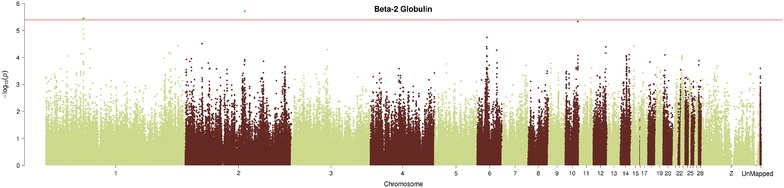
Manhattan plot for the percentage of β-globulin. Manhattan plot of genome-wide −log10 (p values) for the percentage of β-globulin. P values were adjusted using the false-discovery rate (FDR) at a significance level of q value <0.1. SNPs labeled in *green* have q value <0.1

Among the traits measured in the subset of 176 animals, the genomic region located between 52.31 and 52.63 Mb on GGA1 and the SNP AX-76165289 that mapped to GGA2 were significantly associated with the percentage of β2-globulin in the blood plasma (Fig. 4). The genomic region on GGA1 contains 16 SNPs that were associated with the percentage of β2-globulin in blood plasma. Individually, the SNPs on GGA1 explain approximately 13.4 % of the total variance, and SNP AX-76165289 explains 14.2 % of the total variance. The Q–Q plot for β2-globulin also shows that strong associations were observed (See Additional file 9: Figure S3). SNP AX-76165289 is located in the *FHOD3* gene on GGA2, while five SNPs on GGA1 (between 52.31 and 52.63 Mb) are located in the *LARGE* gene (See Additional file 6: Table S6).

### Biological pathway analysis

**Fig. 5:**
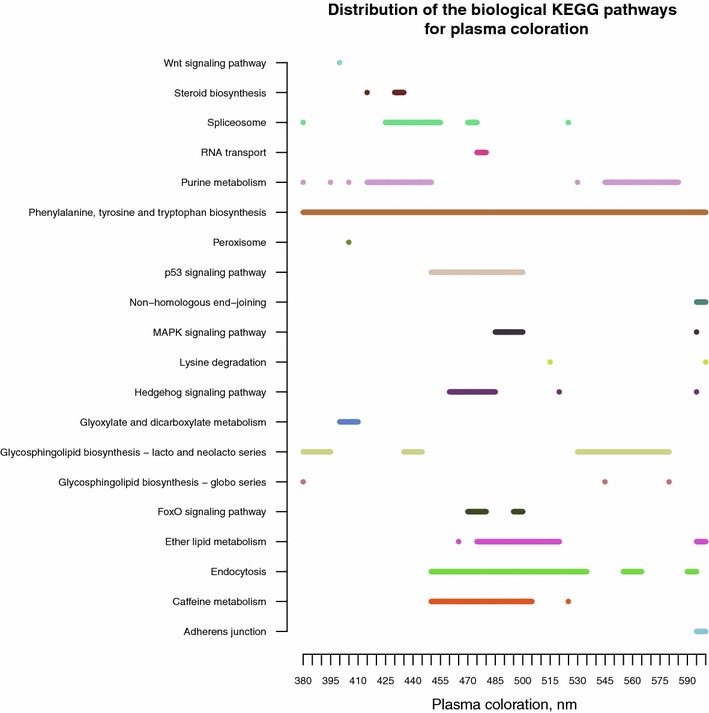
Distribution of the biological pathways that were significantly enriched with genes in genomic regions associated with plasma coloration (PC). Distribution of the biological pathways significantly (P < 0.05) that were enriched with genes in genomic regions associated with plasma measurement for all 45 wavelengths. Coloration of blood plasma was measured as the level of absorbance for 45 wavelengths in the range from 380 to 600 nm. This figure illustrates significant KEGG pathways for PC measurements in the range from 380 to 600 nm

**Fig. 6:**
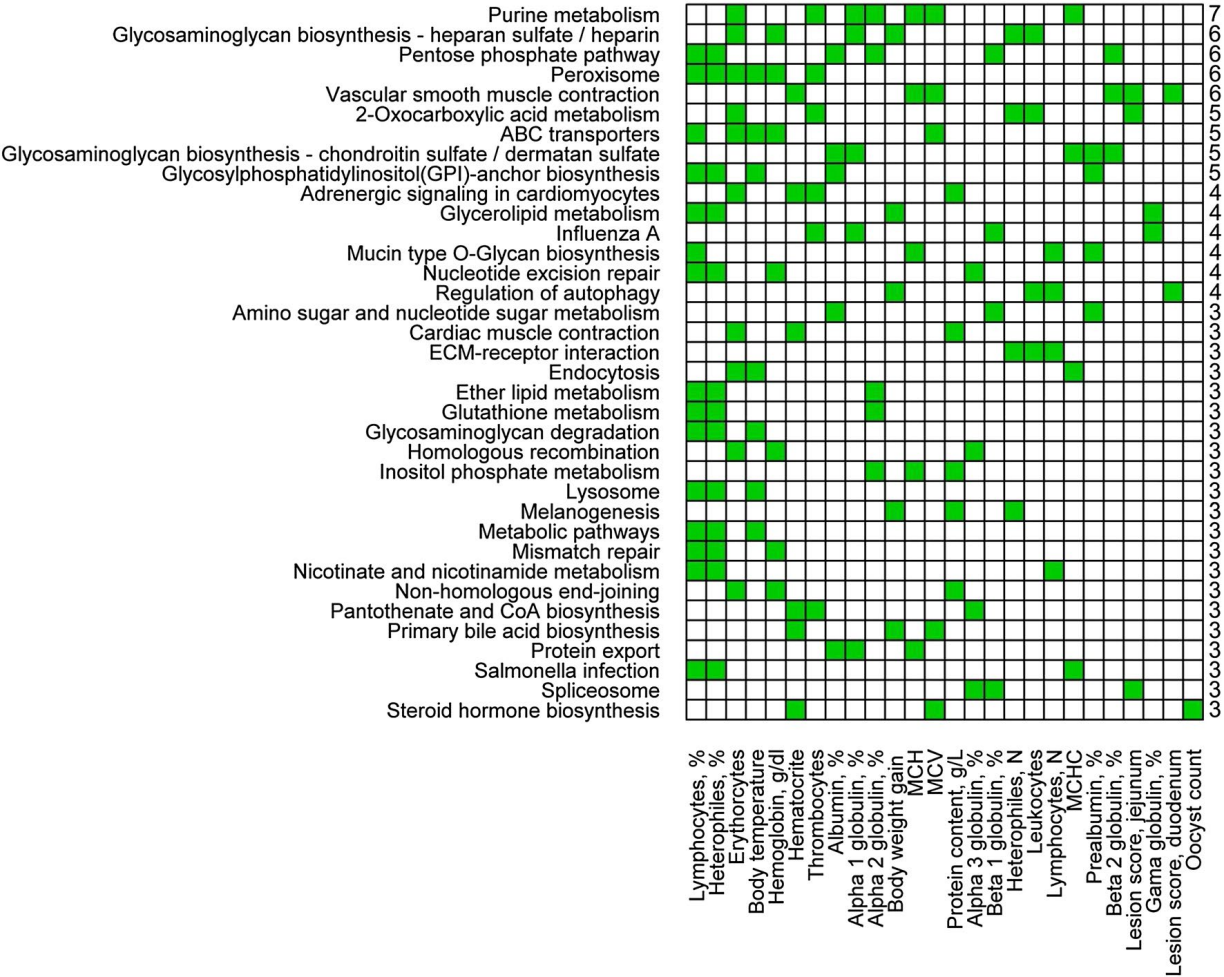
Distribution of the biological pathways that were significantly enriched with genes in genomic regions associated to all measured traits except plasma coloration (PC). Distribution of significantly (P < 0.05) that were enriched with genes in genomic regions associated with all measured traits except plasma coloration (PC). This figure summarizes significant biological pathways that occurred more than three times for measured traits

To conduct the biological pathway analysis, we used all SNPs that were included in the GWAS. These SNPs were mapped to their corresponding genes using the latest genome assembly. This list of SNPs and their corresponding genes was combined with the publicly available biological KEGG pathway database. Finally, we obtained a list containing 52,204 SNPs that were included in the GWAS; these were assigned to 162 of the biological pathways associated with 4342 genes in the chicken genome KEGG PATHWAY repository (see “Methods”). The list of KEGG pathways which were significantly (P < 0.05) enriched with genes in genomic regions associated with the measured traits is in Table S7 (See Additional file 10: Table S7). The distributions of the most frequent significant biological pathways differed considerably according to whether all measured traits or all measured traits except PC measurements were considered (See Additional file 11: Figure S4). Several biological pathways were characteristic of the genomic regions associated with PC measurements (See Additional file 11: Figure S4), and due to the multiple wavelength measurements, they were more frequent, which was not the case when the results were summarized without considering PC measurements (See Additional file 11: Figure S4). For example, the phenylalanine, tyrosine and tryptophan biosynthesis pathway was one of the most frequent pathways when considering only the PC measurement results and was not common for other measured traits [(See Additional file 11: Figure S4) and Fig. 5]. Therefore, we present the PC results (Fig. 5) and all other measured pathways separately (Fig. 6).

For PC measured from 380 to 600 nm, we detected 20 significant biological pathways (See Additional file 10: Table S7). The most frequent significant biological pathways for PC across all measured wavelengths were: phenylalanine, tyrosine and tryptophan biosynthesis, endocytosis, purine metabolism, glycosphingolipid biosynthesis—lacto and neolacto series, ether lipid metabolism, caffeine metabolism, spliceosome, and the p53 signaling pathway (Fig. 5). Each of the mentioned biological pathways occurred more than 10 times as significant when considering all PC measurements (See Additional file 10: Table S7).

In total, seven biological pathways were significantly associated with BWG (See Additional file 10: Table S7). Glycosaminoglycan biosynthesis—heparan sulfate/heparin, glycerolipid metabolism, primary bile acid biosynthesis, melanogenesis, proteasome and regulation of autophagy were the most frequent significantly associated pathways across all measured traits except PC (Fig. 6). In total, 13 and 12 biological pathways were significantly associated with BT and HEMA, respectively (See Additional file 10: Table S7). For BT, the strongest associations were observed with metabolic pathways (P = 0.002) and the ErbB signaling pathway (P = 0.003). For HEMA, the strongest associations were observed with vascular smooth muscle contraction (P < 0.001) and pantothenate and CoA biosynthesis (P = 0.004) (See Additional file 10: Table S7). For duodenal and jejunal LS, five and four biological pathways were significant, respectively (See Additional file 10: Table S7). Among these, vascular smooth muscle contraction and porphyrin and chlorophyll metabolism were significantly enriched for both duodenal and jejunal LS (See Additional file 10: Table S7).

Considering the long list of measured traits for BC and PPP, we summarize the most interesting results. Regarding the percentage of lymphocytes and heterophils, we observed 15 and 13 significant biological pathways, respectively, with 13 pathways being common for both traits (Fig. 6). Furthermore, the percentages of lymphocytes and heterophils were associated with the largest number of significant biological pathways (Fig. 6). Regarding the number of thrombocytes and erythrocytes, 13 and 10 significant biological pathways were observed, respectively (See Additional file 10: Table S7), and these two traits had four significant pathways in common (Fig. 6). Regarding PPP, both the percentages of α1-globulin and α2-globulin were associated with 10 significant biological pathways (See Additional file 10: Table S7). Percentages of prealbumin and albumin shared the following significant pathways: amino sugar and nucleotide sugar metabolism, glycosaminoglycan biosynthesis—chondroitin sulfate/dermatan sulfate and glycosylphosphatidylinositol (GPI)-anchor biosynthesis (Fig. 6).

The three most frequent significant pathways, considering all measured traits, including PC, were phenylalanine, tyrosine and tryptophan biosynthesis, endocytosis and purine metabolism (See Additional file 11: Figure S4). The most frequent significant pathways, considering all traits except PC, included purine metabolism, glycosaminoglycan biosynthesis—heparan sulfate/heparin, the pentose phosphate pathway, and peroxisome and vascular smooth muscle contraction (Fig. 6) and (See Additional file 11: Figure S4).

### Network-based analysis

**Fig. 7:**
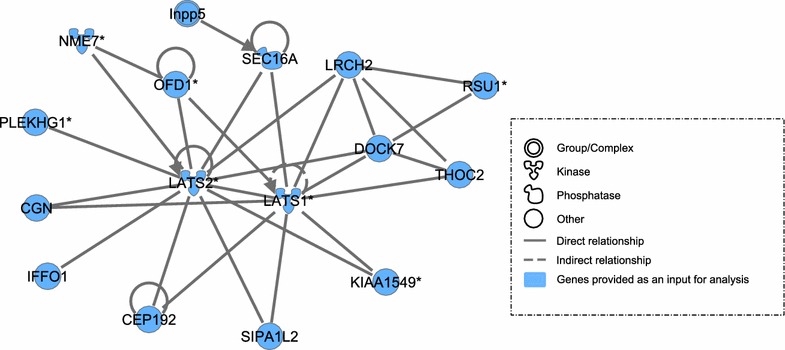
Network of interactions between GWAS candidate genes using ingenuity pathway analysis (IPA). The network shows molecular interactions between the products of the candidate genes enriched for significantly associated SNPs (P < 10^−4^) with large tumor suppressor kinases 1 (LATS1) and 2 (LATS2) as the most interesting candidates. Relationships were determined using information contained in the IPA repository. The *blue label* indicates the genes that were enriched for significantly associated SNPs. The network was obtained using the global gene list determined by combining gene lists for all traits

**Fig. 8:**
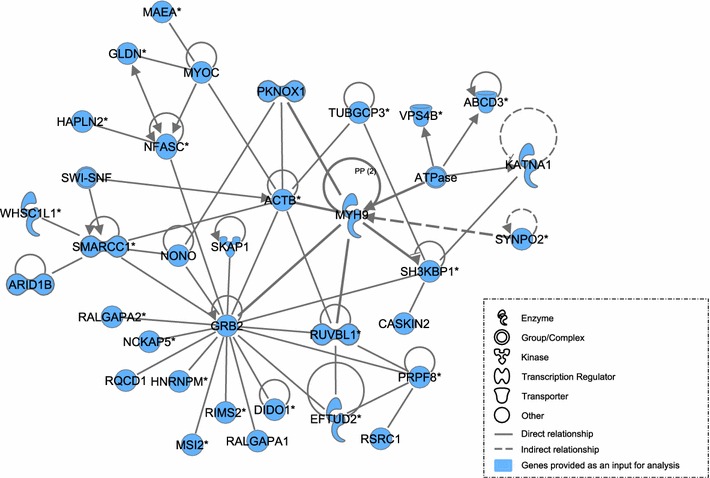
Network of interactions between GWAS candidate genes using ingenuity pathway analysis (IPA). The network shows molecular interactions between the products of the candidate genes enriched for significantly associated SNPs (P < 10^−4^) with myosin-6 (MYH6) as the most interesting candidate. Relationships were determined using information contained in the IPA repository. The *blue label* indicates the genes that were enriched for significantly associated SNPs. The network was obtained using the global gene list determined by combining gene lists for all traits

The network-based analysis was performed using the ingenuity pathway analysis (IPA) tool. Gene lists used as input files for the IPA tool were obtained by extracting genes containing SNPs, with an inferred genome-wide significance level (P < 10^−4^) for all QTL. The IPA tool produced 76 putative networks using 27 gene lists. The majority of networks were related to general molecular and cellular processes such as cell to cell signaling, nucleic acid metabolism and replication, cell cycle, with several of the networks involved in more specific pathways such as gastrointestinal diseases and organismal injury and abnormalities. In the context of the large-scale challenge study, the most informative networks were the network of interacting molecules grouped around large tumor suppressor kinases 1 (LATS1) and 2 (LATS2) with IPA score 41 (Fig. 7) and the second network with myosin heavy chain 6 (MYH6) in the center with IPA score 51 (Fig. 8).

## Discussion

Identifying genomic regions that underlie the response to *Eimeria maxima* in broilers allows us to understand the associated molecular mechanisms and provides us with candidate genes and genomic regions that can be used in breeding for improved resistance to coccidiosis. The host response to *Eimeria* is a complex trait controlled by a wide range of biological processes, which are in turn controlled by many genes with a small effect and a small number of genes with a moderate or large effect. Several QTL regions that are significantly associated with traits measured after the response to *Eimeria* challenge were detected in F_2_ crosses obtained from experimental lines with different degrees of susceptibility to coccidiosis [16, 17, 36]. In addition, similar approaches have been used for the identification of genomic regions associated with innate and adaptive immunity in laying hens [37] as well as survival rate and aimed at improving general robustness, especially in laying hens [38]. In addition, information regarding the genomic regions that are strongly associated with desirable traits can be incorporated in commercial poultry breeding programs. Regarding broilers, haplotypes that are associated with desirable traits identified in the final product of the four-crossway breeding scheme can be traced back in pedigreed populations for which selection would be performed within the pure lines. This approach may potentially improve general innate immunity as well as resistance to specific pathogens such as *Eimeria* species.

However, a more detailed understanding of the genetic mechanisms that control the response to *Eimeria* infection, as in the case of all other complex traits, requires a sufficiently large sample size, dense SNP coverage that can exploit the linkage disequilibrium and informative phenotypes [39]. Therefore, we performed the large-scale challenge study on 1936 commercial Cobb500 animals, which were genotyped using the 580K Affymetrix^®^ Axiom^®^ high-density genotyping array, providing considerable statistical power for GWAS for traits measured on all 1936 animals. Taking advantage of the size and structure of the challenge experiment [19], we conducted a GWAS to obtain more information on the underlying genetic determinism of the response to *E. maxima* in broilers. In addition, a post-GWAS functional analysis was performed to further understand the biology of the response to *E. maxima* in broilers.

### GWAS

Previous QTL studies have reported that several genomic regions are associated with BWG in response to coccidiosis [15, 20]. Comparing our results with these previous studies, only one highly significant SNP that overlaps with a QTL on GGA3 (between 263 and 282cM; 98.1 and 107.0 Mb), detected by Pinard-van der Laan et al. [16] and Bacciu et al. [17], was observed. However, these different results were not completely unexpected considering that the previously conducted challenge study was performed with animals at a different age and that originated from very different experimental lines.

The *MGAT4C* gene is close to the significantly associated genomic region on GGA1. This gene encodes a glycosyltransferase that is involved in the transfer of *N*-acetylglucosamine (GlcNAc) to the core mannose residues of N-linked glycans, also known as N-linked glycosylation. N-linked glycosylation has been shown to be essential to HIV-1 pathogenesis [40]. Furthermore, there is a wide range of well-described disorders that affect primarily N-glycan assembly, with several including gastrointestinal disorders [41]. In addition, a study on humans showed a strong association between the apolipoprotein B level and SNP variants in the *MGAT4C* gene [42].

The *KCNK3* gene is the closest gene to the GGA3 genomic region, which was significantly associated with BWG. The *KCNK3* gene encodes a member of the potassium channel superfamily, which has been associated with pulmonary hypertension in humans [43]. Broilers are known to suffer from cardiovascular disorders [44, 45], which may be related to the *KCNK3* gene. Furthermore, this gene may be associated with the general robustness of broilers and their ability to cope with stress induced by the *E. maxima* challenge.

The *THBS1* gene is located upstream of the significantly associated region GGA5 [between 28.95 and 29.11 Mb, (See Additional file 6: Table S6)]. In addition, we also analyzed putative GENSCAN Gene Predictions that are supported by a few spliced EST (See Additional file 12: Figure S5); however, no similarity was observed with known proteins, which indicates that *THBS1* was a better candidate. Human THBS1 is involved in the regulation of angiogenesis and tumorigenesis in healthy tissues and cell adhesion [46, 47]. Furthermore, in the study by Heams et al. [18], *THBS1* was among the top 10 of 1473 significantly differentially expressed genes in the caecum between Fayoumi (resistant) and Leghorn (susceptible) animals infected with *E. tenella*. Moreover, a porcine transcriptome study showed that *THBS1* is strongly repressed in the in vitro stimulation of porcine peripheral-blood mononuclear cells (PBMC) with tetradecanoyl phorbol acetate (TPA)/ionomycin [48]. Finally, a recent study showed that human THBS1 plays an important role in the innate immune response during respiratory bacterial infection [49], which may be of interest regarding *Eimeria* infection. Based on these observations, *THBS1* is a good candidate gene for further functional studies.

Regarding PC, our GWAS results show little overlap with previous QTL mapping studies [16]. We observed the strongest signal with the SNP in the *MAN2C1* gene (See Additional file 6: Table S6) in association with PC measured in the range from 465 to 510 nm. The associated SNP is described as a non-synonymous polymorphism (http://www.ncbi.nlm.nih.gov/projects/SNP/snp_ref.cgi?rs=314637018). The *MAN2C1* gene encodes the α-mannosidase class 2C enzyme, which is one of the key enzymes involved in N-glycan degradation [50]. Recent studies have indicated that *MAN2C1* expression is crucial for maintaining efficient protein N-glycosylation [51] as well as cell–cell adhesion [52]. Glycans are important molecules in numerous essential biological processes, including cell adhesion, molecular trafficking and clearance, receptor activation, signal transduction, and endocytosis [53]. In contrast, changes in the PC reflect the status of intestinal absorption, changes in the production of protein carriers and their antioxidant effect in response to *Eimeria* infection [54] and reflect several of the processes that MAN2C1 may impact. Furthermore, several significant glycol-related pathways were significantly enriched in the biological pathway analysis for PC measurements (Fig. 5).

Regarding the percentage of β-globulins, we identified *FHOD3* and *LARGE* as potential candidate genes (See Additional file 6: Table S6). *FHOD3* encodes a protein that is a member of a formin subfamily and is involved in the regulation of cell actin dynamics [55]. However, how FHOD3 may be involved in the regulation of plasma β-globulin levels is difficult to discern because the current knowledge regarding FHOD3 is scarce and restricted to human and mouse functional studies [56, 57]. The *LARGE* gene encodes an enzyme glycosyltransferase that is involved in alpha-dystroglycan glycosylation and is capable of synthesizing glycoprotein and glycosphingolipid sugar chains [58]. The exact function of LARGE is not fully known; however, mutations in the human *LARGE* gene have been described to cause congenital muscular dystrophy type 1D (MDC1D) [59].

Taking all measured traits into account, we identified 22 highly associated SNPs; however, GWAS was performed on two sets of traits: the first set of traits was measured on all 2024 animals, and the second set was measured on the subset of 176 animals, for which the statistical power to detect potential candidate genes differed due to the different sample sizes. Regarding BWG and PC that were measured on all animals, two functionally well-supported candidate genes (*THBS1* and *MAN2C1*) were detected. However, we did not identify any candidate regions for BT and HEMA, potentially because these traits have not been as affected in challenged animals compared with PC and BWG [19]. Furthermore, an infection caused by *E. maxima* is often characterized by intestinal malabsorption and not by severe bloody diarrhoea, which is the case with *E. tenella*. This finding further indicates that HEMA is not very informative when measuring the response to *E. maxima* infection [19]. Similarly, BT is difficult to interpret with respect to *Eimeria* infection because this trait may be influenced by other factors as discussed by Hamzic et al. 2015 [19]. However, we have not identified many candidate genes or genomic regions in the GWAS performed by using traits measured in the 176 animals, which may be primarily due to the sample size, which was 10 times smaller in comparison to the traits measured on all animals, and to the complexity of the genetic parameters that control these traits. In addition, this absence of identifiable candidate genes may be partially due to the precision of measured traits and rather stringent significance thresholds used in GWAS. Therefore, we performed biological pathway analysis, which enabled an increase in the statistical power to detect significant association. An increase in the statistical power is possible because we decreased the number of statistical tests performed by compressing individual SNPs into biological pathways [60].

The final aim is to transfer the acquired knowledge to the poultry breeding industry. The identified candidate genes and genomic regions can be used in breeding for improved resistance to coccidiosis. The primary obstacle in achieving this goal is relating the knowledge regarding the candidate genes identified in the final product (Cobb500) with the grand-parent pure lines where the actual selection is performed. This task becomes rather complex, considering that the poultry populations and the crossing design are not in the public domain, and due to the proprietary nature of information regarding the grandparent lines. Future studies will identify the best approach to trace the identified regions to the grandparent lines.

### Biological pathway analysis

The biological pathway studies based on the GWAS results can potentially extend the knowledge obtained from GWAS studies by identifying the cumulative effect of gene sets [61]. Furthermore, understanding the biological pathways increases the power to detect statistically significant associations because fewer statistical tests are performed as a consequence of assigning individual SNPs to the respective biological pathways. Therefore, the number of tests is decreased from over 400,000 (number of SNPs) to approximately 160 (number of pathways) using a priori biological information. For this purpose, we assigned genes, containing SNPs used in GWAS, to the biological KEGG pathways. Therefore, biological KEGG pathways are considered as a set of genes that have been used for further analysis. However, we have to be aware that the biological pathway analysis should still be primarily viewed as an exploratory technique because the current statistical methodologies used for gene set/pathway analysis need further development [61]. Moreover, the available biological pathway databases necessary for this kind of analysis are not completely annotated and do not contain all the genes present in the chicken genome. In this study, we used a statistical modeling methodology that assesses the cumulative effect of sets of SNPs on the measured traits as presented by Jensen et al. [31] and Buitenhuis et al. [62]. For this purpose, we succeeded in assigning 52,204 SNPs from GWAS to 4342 annotated genes in the chicken genome.

For PC, the top four most frequently affected pathways across all wavelengths include phenylalanine, tyrosine and tryptophan biosynthesis, endocytosis, purine metabolism, and glycosphingolipid biosynthesis—lacto and neolacto series (Fig. 5).

Phenylalanine, tyrosine and tryptophan biosynthesis is the most commonly affected pathway when only PC is considered (Fig. 5) and (See Additional file 11: Figure S4). Phenylalanine, tyrosine and tryptophan have important roles in the regulation of the immune response [63]. Phenylalanine is indirectly involved in the regulation of nitric oxide (NO) synthesis [64], and NO is known to have multiple roles related to the immune response such as signaling properties, regulating cytokine production and killing pathogens [65]. Tyrosine is used as a precursor for the production of dopamine, catecholamines and melanin. Dopamine is a neurotransmitter known to be involved in the regulation of immune response, and melanin has antioxidant properties [63]. In addition, interferon gamma (IFN-γ) suppresses the growth of *Toxoplasma gondii* through the intracellular depletion of tryptophan [66]. Deprivation of tryptophan produces a deleterious effect on *Toxoplasma gondii* replication. *Toxoplasma gondii* belongs to the same order (*Eucoccidiorida*) of intracellular single-cell parasites as *E. maxima*. Furthermore, Laurent et al. [67] reported a strong increase in IFN-γ mRNA expression in chickens infected with *Eimeria* spp. Based on this finding and the results from the biological pathway analysis, tryptophan depletion may also be involved in the innate immune response during *E. maxima* infection.

The second most commonly affected pathway when considering PC is the purine metabolism pathway (Fig. 5) and (See Additional file 11: Figure S4). This pathway regulates nucleotide metabolism and is important for successful cell division. In addition, we observed that the purine metabolism pathway is significantly associated with erythrocyte number, mean corpuscular hemoglobin (MCH), mean corpuscular hemoglobin concentration (MCHC) and mean cellular volume (MCV) (Fig. 6). This association may be explained by an increased demand for cell division of blood cell progenitors and the regeneration of the intestinal epithelium due to the effects of the infection because the *E. maxima* infection has been characterized by severe lesions of the intestinal lining in broilers [19].

Endocytosis has been shown to play an important role in both innate and adaptive immune responses [68], which may explain why endocytosis may be among the most commonly affected pathways when PC measurements are considered (Fig. 5). Finally, glycosphingolipid biosynthesis—lacto and neolacto series, like other glycol-related pathways (Fig. 5), is involved in the production of glycoconjugate receptors, which are used by microbes to enter the host cell and are of critical importance in the early stage of the innate immune response [69, 70]. Moreover, similar paths of the host cell invasion have been previously described in the cases of *Eimeria* and *Toxoplasma* [71, 72].

We also summarized the frequency of the most common significant biological pathways excluding PC (Fig. 6). In this case, glycosaminoglycan biosynthesis involving heparan sulfate/heparin, the pentose phosphate pathway and the peroxisome are the most frequent significantly enriched biological pathways. The pentose phosphate pathway is a metabolic pathway that regulates the production of nicotinamide adenine dinucleotide phosphate (*NADPH*) and pentose, which are essential for the synthesis of nucleic and ribonucleic acids, respectively. The pentose phosphate pathway seems to play a role in plasma protein component and heterophil and lymphocyte production, which may be explained as a response to the increased production of these components during the primary response to the challenge (Fig. 6) [73]. The peroxisome pathway controls the metabolism of reactive oxygen components, which are known to be toxic to bacteria and several parasites and play a significant role in resilience and immunity to infectious diseases [73]. Moreover, the peroxisome pathway was significantly associated with blood components such as number of erythrocytes and percentage of heterophils and lymphocytes (Fig. 6).

These findings demonstrate that the response to *Eimeria* infection is characterized by a strong effect on essential metabolic pathways as well as innate immune response-related pathways. Among the essential metabolic pathways, the most frequently affected are phenylalanine, tyrosine and tryptophan biosynthesis, purine metabolism and the pentose phosphate pathway. In addition, the most frequent innate immune response-related pathways are glycol-related pathways, the peroxisome pathway and the endocytosis pathway.

### Network-based analysis

The network-based analysis was performed as a complementary approach to the biological pathway analysis to build gene networks associated with responses to the *E. maxima* challenge using bibliography-based proven relationships that are available through the Ingenuity Pathway repository. The network-based analysis was performed independently from the biological pathway analysis and was based on a list of genes enriched for SNPs that are associated (p < 10^−4^) with the traits measured during the *E. maxima* challenge (See Additional file 4: Table S4). The network-based analysis approach, implemented in the IPA tool, assumes that genes used as an input interact with each other, and these interactions are reconstructed based on the relationships shown in the literature [34]. The network-based analysis aims at exploring the cumulative effect of sets of genes that individually explain a moderate part of the variation for a measured trait and that cannot be identified during GWAS when the strict significance threshold was applied.

Figure 7 illustrates the network formed by several of the molecules grouped around LATS1 and LATS2 with multiple direct connections with other molecules enriched with significantly associated SNPs. LATS1 and LATS2 are known to be involved in the regulation of intestinal epithelium renewal [74], which may be explained by intensified tissue repair upon *E. maxima* challenge. In addition, phenylalanine, tyrosine and tryptophan biosynthesis, purine metabolism and the pentose phosphate pathway are the most frequent significant pathways associated with all measured traits in the KEGG biological pathway analysis. All three of these KEGG pathways are associated with increased DNA replication, cell metabolism and protein degradation, which are essential during the tissue repair process.

The second network has the MYH6 as a key molecule connected with several direct significant relationships to the other genes that are enriched for significantly associated SNPs (Fig. 8). The *MYH6* gene encodes the alpha heavy chain subunit of cardiac myosin. In mice, inactivation of the specific mutant *MYH6* transcript suppresses hypertrophic cardiomyopathy [75]. In addition, we also identified *KCKN3*, which is associated with pulmonary hypertension, as one of the candidate genes because heart failure and ascites have been well documented in broiler chickens [44, 45]. The primary reason for these problems can be attributed to an intensive selection in poultry breeding during the last 60 years [76]. Therefore, we can potentially indicate which animals are able to maintain a normal function of the cardiovascular system and have an advantage in the face of *Eimeria* infection.

Based on the post-GWAS functional analysis, the broiler response to *E. maxima* is centered on tissue repair and recovery, general robustness and maintenance of tissue integrity, restoring intestinal homeostasis after the challenge. The described processes, which may bring a comparative advantage in the broiler’s ability to cope with the challenge, can be described as resilience to acute *Eimeria* infection. In contrast to the previously conducted studies [16, 17], which reported associations with genes involved in the immune response, we primarily observed associations with genes, biological pathways and gene networks that are involved in tissue repair and recovery and tissue integrity maintenance. However, previous studies [16, 17] were conducted on experimental layer populations challenged with *E. tenella* at 28 days of age. In regard to this, broilers are also able to establish complete immunity 16 days after being challenged with *E. maxima* [77]. Therefore, one would assume that this challenge study would identify genetic variants associated with processes related to immune responses, which did not occur in this study and may be due to the effect of the infection doses (50,000 oocysts), which were optimized to produce severe clinical signs as reported by Hamzic et al. [19]. We argue that the more resilient animals are able to maintain their biological homeostasis and manage the consequences of the infection, which, in the context of this challenge, exceeded the importance of building an adequate immune response.

## Conclusions

We identified 22 SNPs significantly associated with four different traits at q value <0.1. Two candidate genes, *MAN2C1* and *FHOD3*, were significantly associated with PC measured in the range from 465 to 510 nm and the percentage of β2-globulin in blood plasma, respectively. Moreover, we identified three genomic regions on GGA1 (*MGAT4C*), GGA3 (*KCNK3*) and GGA5 (*THBS1*) that are significantly associated with body weight gain and the percentage of β2-globulin.

The post-GWAS functional analysis, which combined two independent approaches (the biological pathway analysis and network-based analysis), indicated that the genes and biological pathways involved in tissue repair, general robustness as well as the primary immune response may play an important role during the primary stage of *E. maxima* infection in broilers.

Studies that focus on the transfer of the acquired knowledge to poultry breeding considering the specificities of the broiler breeding scheme are currently under way. Finally, a follow-up transcriptome study is ongoing, which aims at integrating results from the GWAS study and further investigation of the genetic mechanisms that control the response to *Eimeria* infection in broilers.

## Availability of supporting data

No new SNPs were discovered in the preparation of this manuscript. The SNPs used in this manuscript are from the chicken 580 K Affymetrix^®^ Axiom^®^ HD genotyping array: http://media.affymetrix.com/support/technical/datasheets/axiom_chicken_array_plate_datasheet.pdf. SNP names and location can be found at http://www.affymetrix.com/catalog/prod670010/AFFY/Axiom%26%23174%3B+Genome%26%2345%3BWide+Chicken+Genotyping+Array#1_3.

## Additional files

10.1186/s12711-015-0170-0 Title: Quality control of genotype data. Description: The genotype data were obtained from 1972 animals that were alive at day 16 of the challenge study. Genotype data were produced using 580 K Affymetrix^®^ Axiom^®^ HD genotyping array. The call rate threshold was set at 98 %, and all SNPs and samples with values less than 98 % were excluded. The minor allele frequency threshold was set at 1 % and animals with values less than 1 % were excluded. Heterozygote excess was assessed based on the distribution of F-statistics adjusted for skewness using adjbox function from the R package robustbase. Control animals were also excluded from the analysis. A total of 138,568 SNPs out of 580,961 were removed during the quality control.
10.1186/s12711-015-0170-0 Title: Descriptive statistics for the distances between SNPs across chromosomes. Description: The table contains information on the number of SNPs per chromosome and the mean, median and standard deviation (SD) for the average distances between neighboring SNPs. Results in the table refer to genotype data after quality control.
10.1186/s12711-015-0170-0 Title: List of annotated SNPs used for Biological Pathway Analysis. Description: The table contains SNPs used in the genome-wide association study with p-values < 10^−4^ with the corresponding information on the genes in which they are located. Abbreviations used for column names: RS ID: Reference SNP ID number, SYMBOL: HGNC symbol for gene, NCBI GENE ID: NCBI gene ID, CHR: chromosome, CHR POS: chromosome position, GENE REGION: gene region of the corresponding gene, SS ID: submitted SNP ID, AFFYMETRIX: Affymetrix SNP ID, ALLELE: SNP alleles. The remaining columns are p-values for each trait with column names corresponding to trait abbreviations used in the manuscript.
10.1186/s12711-015-0170-0 Title: List of annotated SNPs and their corresponding genes used for Ingenuity Pathway Analysis. Description: The table contains SNPs with the corresponding information on the genes in which they are located and together with p-values obtained in the genome-wide association study.
10.1186/s12711-015-0170-0 Title: Descriptive statistics of the measured traits. Description: Descriptive statistics of the traits measured on the challenged animals during the large-scale challenge study. Plasma coloration was measured as absorbance (optical density) of blood plasma at different wavelengths. Oocyst count is expressed as the raw number of oocysts per gram of feces. Erythrocyte count is expressed in millions per mm^3^ (M∙mm^−3^). White blood cell and thrombocyte count is expressed in absolute number per mm^3^ (count∙mm^−3^. Lymphocytes and heterophils are also expressed in the percentage of total number of white blood cells. MCV, MCH and MCHC correspond to mean cellular volume, mean corpuscular hemoglobin and mean corpuscular hemoglobin concentration, respectively.
10.1186/s12711-015-0170-0 Title: Significant SNPs associated with traits measured on all challenged animals. Description: Significant SNPs associated with traits measured on all challenged animals. A genome-wide association analysis was performed using Cobb500 broilers for all the measured traits during the large-scale study. SNPs were considered significant at q-value  < 0.10 using False Discovery Rate adjustment.
10.1186/s12711-015-0170-0 Title: Quantile–quantile plot for body weight gain. Description: Quantile–quantile plot for the body weight gain test statistics.
10.1186/s12711-015-0170-0 Title: Quantile–quantile plot for plasma coloration (485 nm). Description: Quantile–quantile plot for plasma coloration (485 nm) test statistics.
10.1186/s12711-015-0170-0 Title: Quantile–quantile plot for the percentage of β2-globulin. Description: Quantile–quantile plot for β2-globulin test statistics.
10.1186/s12711-015-0170-0 Title: List of biological pathways significantly enriched with genes in genomic regions associated with measured traits. Description: Results for the biological pathway significantly (P < 0.05) enriched with genes in genomic regions associated with measured traits during the large-scale challenge study. The p-values provided in the table represent values for the one-sided test for overlapping SNPs after 1000 permutations.
10.1186/s12711-015-0170-0 Title: Distribution of the most frequent significant biological pathways. Description: Distribution of the most frequent biological pathways being significantly enriched with genes in genomic regions associated with measured traits during the large-scale challenge study. The distribution is considerably different when all measured traits are considered together and when PC measurements are excluded.
10.1186/s12711-015-0170-0 Title: Illustration of the genomic region between 28.95 and 29.11 Mb on GGA5. Description: The genomic region between 28.95 and 29.11 Mb contains three SNPs that were significantly associated with body weight gain (BWG) and also contains several chicken spliced mRNA and EST, which may indicate the presence of a non-annotated chicken gene. Illustration was obtained from the UCSC Genome Browser (ICGSC Gallus_gallus-4.0/galGal4).

## Abbreviations

BWG: body weight gain
HEMA: hematocrit
LS: lesion score
OC: oocyst count
PC: plasma coloration
GWAS: genome-wide association study
QTL: quantitative trait loci
PPP: plasma protein profiles
BC: blood composition
NO: nitric oxide
IFN-γ: interferon gamma
EST: expressed sequence tags
PBMC: peripheral blood mononuclear cell
IPA: Ingenuity Pathway Analysis
HGNC: Human Genome Gene Nomenclature Committee
TPA: tetradecanoyl phorbol acetate
MDC1D: congenital muscular dystrophy type 1D
MCH: mean corpuscular hemoglobin
MCV: mean corpuscular volume
MCHC: mean corpuscular hemoglobin concentration
HEMA: hematocrit level
BT: body temperature
BW: body weight
