# Supplementary figures and images for: Genome-wide association study and biological pathway analysis of the Eimeria maxima response in broilers
Source: Genet Sel Evol. 2015 Nov 25;47:91. doi: 10.1186/s12711-015-0170-0 (PMC4659166; doi:10.1186/s12711-015-0170-0)

# Body Weight Gain

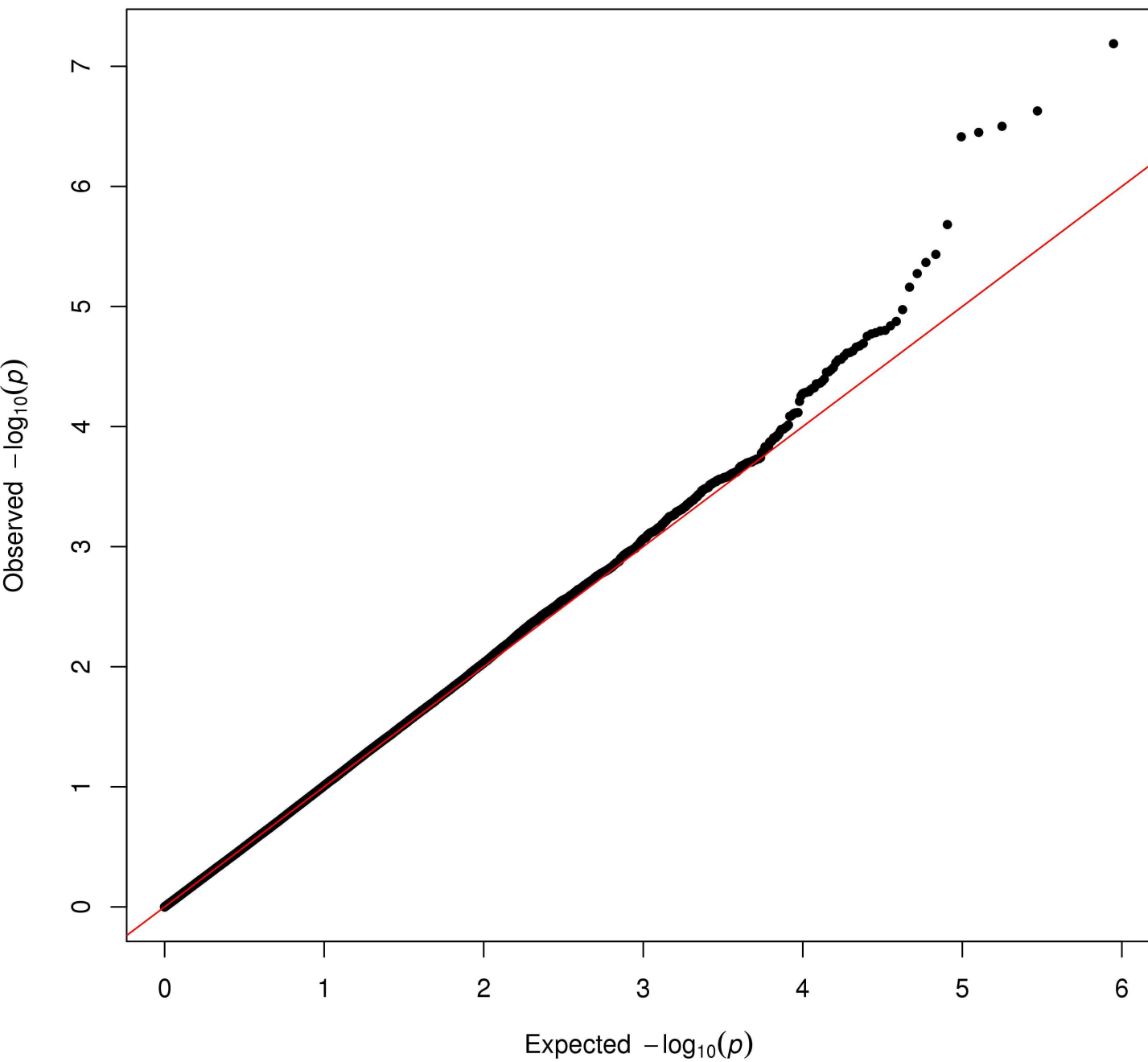

Supplement: Supplementary file 7 — 10.1186/s12711-015-0170-0 Title: Quantile–quantile plot for body weight gain. Description: Quantile–quantile plot for the body weight gain test statistics. [file 12711_2015_170_MOESM7_ESM.pdf]

# Plasma Coloration (485 nm)

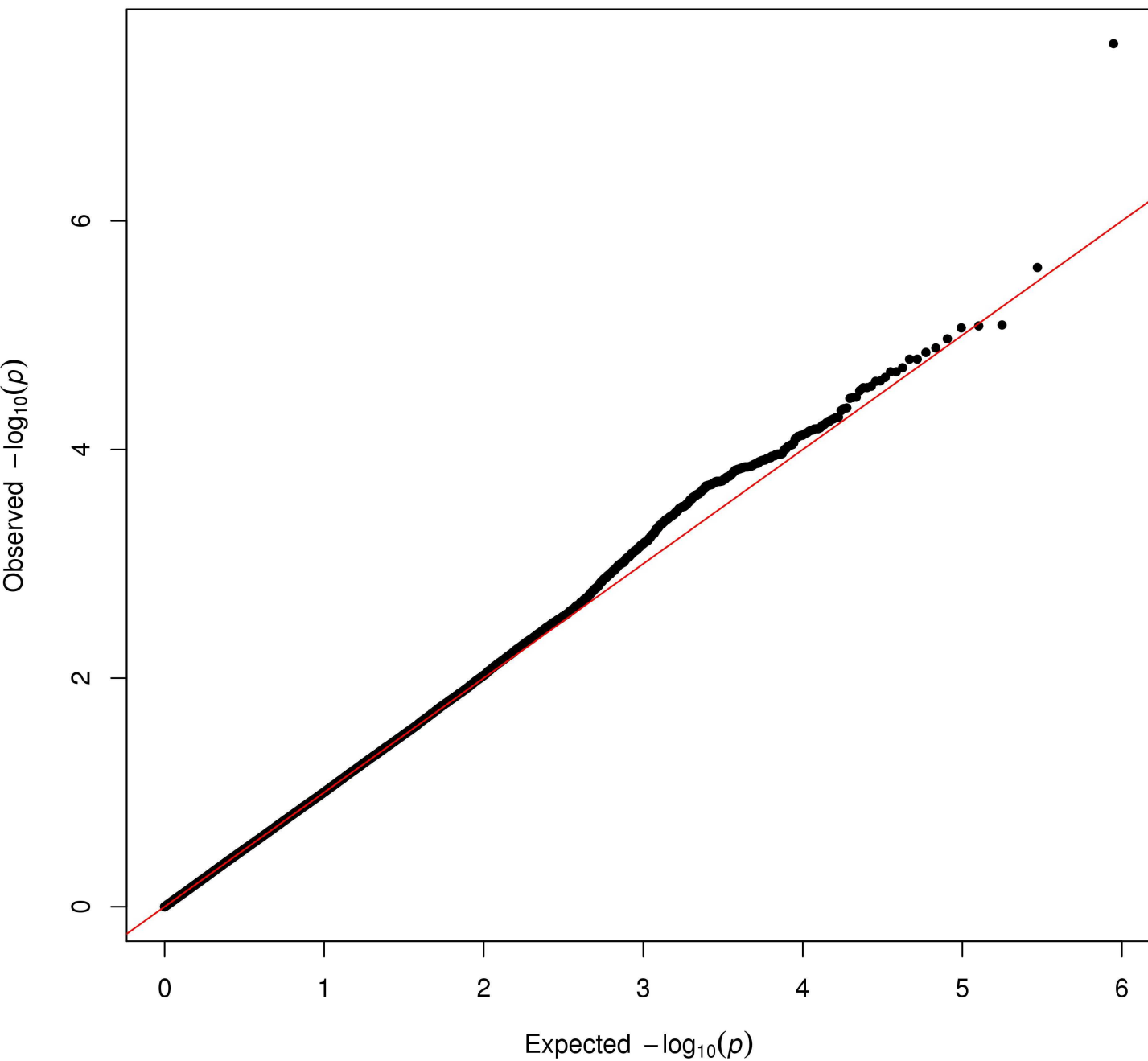

Supplement: Supplementary file 8 — 10.1186/s12711-015-0170-0 Title: Quantile–quantile plot for plasma coloration (485 nm). Description: Quantile–quantile plot for plasma coloration (485 nm) test statistics. [file 12711_2015_170_MOESM8_ESM.pdf]

# Beta-2 Globulin

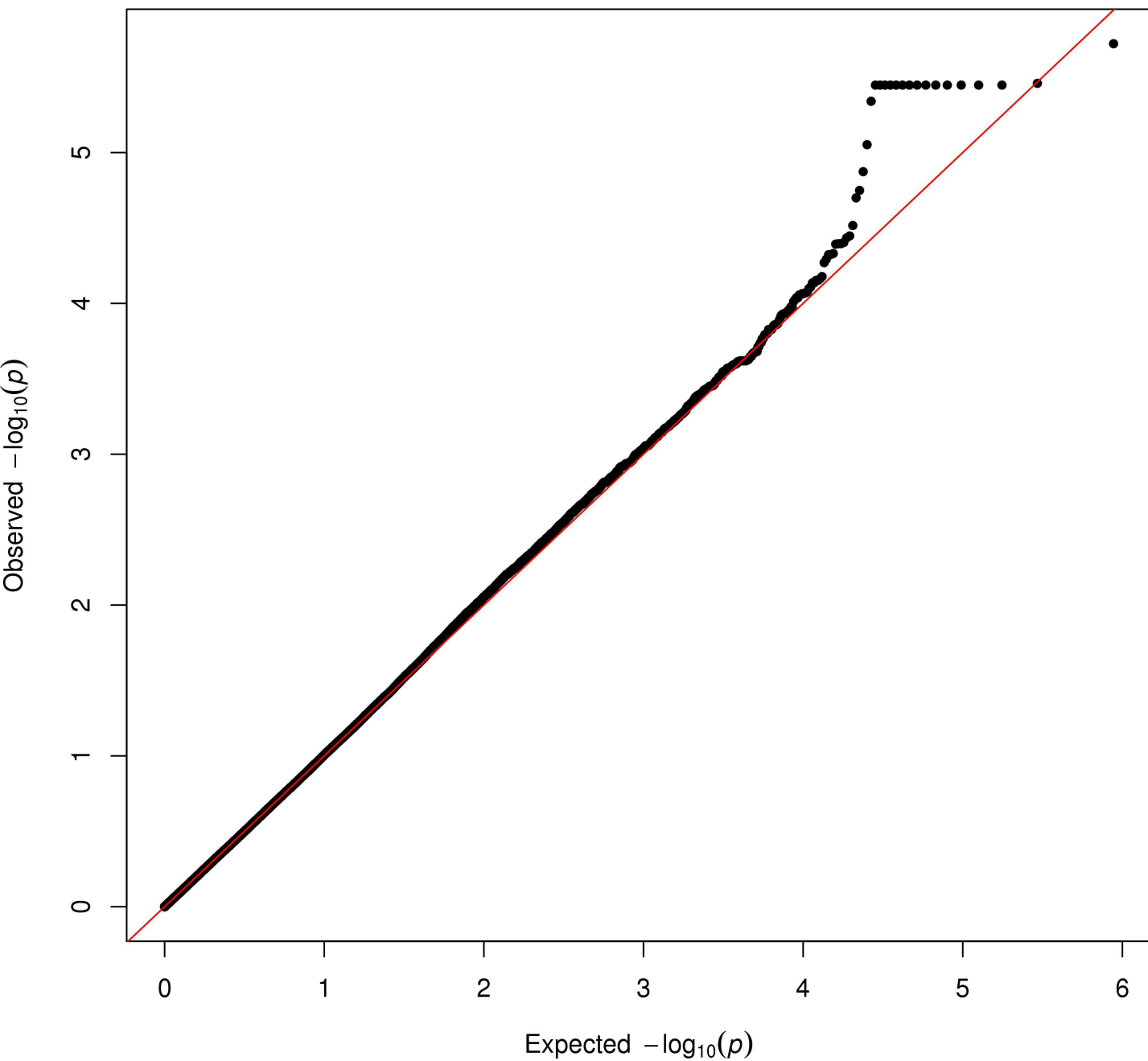

Supplement: Supplementary file 9 — 10.1186/s12711-015-0170-0 Title: Quantile–quantile plot for the percentage of β2-globulin. Description: Quantile–quantile plot for β2-globulin test statistics. [file 12711_2015_170_MOESM9_ESM.pdf]
