# Supplementary material for: Genome-wide association study and biological pathway analysis of the Eimeria maxima response in broilers
Source: Genet Sel Evol. 2015 Nov 25;47:91. doi: 10.1186/s12711-015-0170-0 (PMC4659166; doi:10.1186/s12711-015-0170-0)
Supplement: Supplementary file 11 — 10.1186/s12711-015-0170-0 Title: Distribution of the most frequent significant biological pathways. Description: Distribution of the most frequent biological pathways being significantly enriched with genes in genomic regions associated with measured traits during the large-scale challenge study. The distribution is considerably different when all measured traits are considered together and when PC measurements are excluded. [file 12711_2015_170_MOESM11_ESM.pdf]

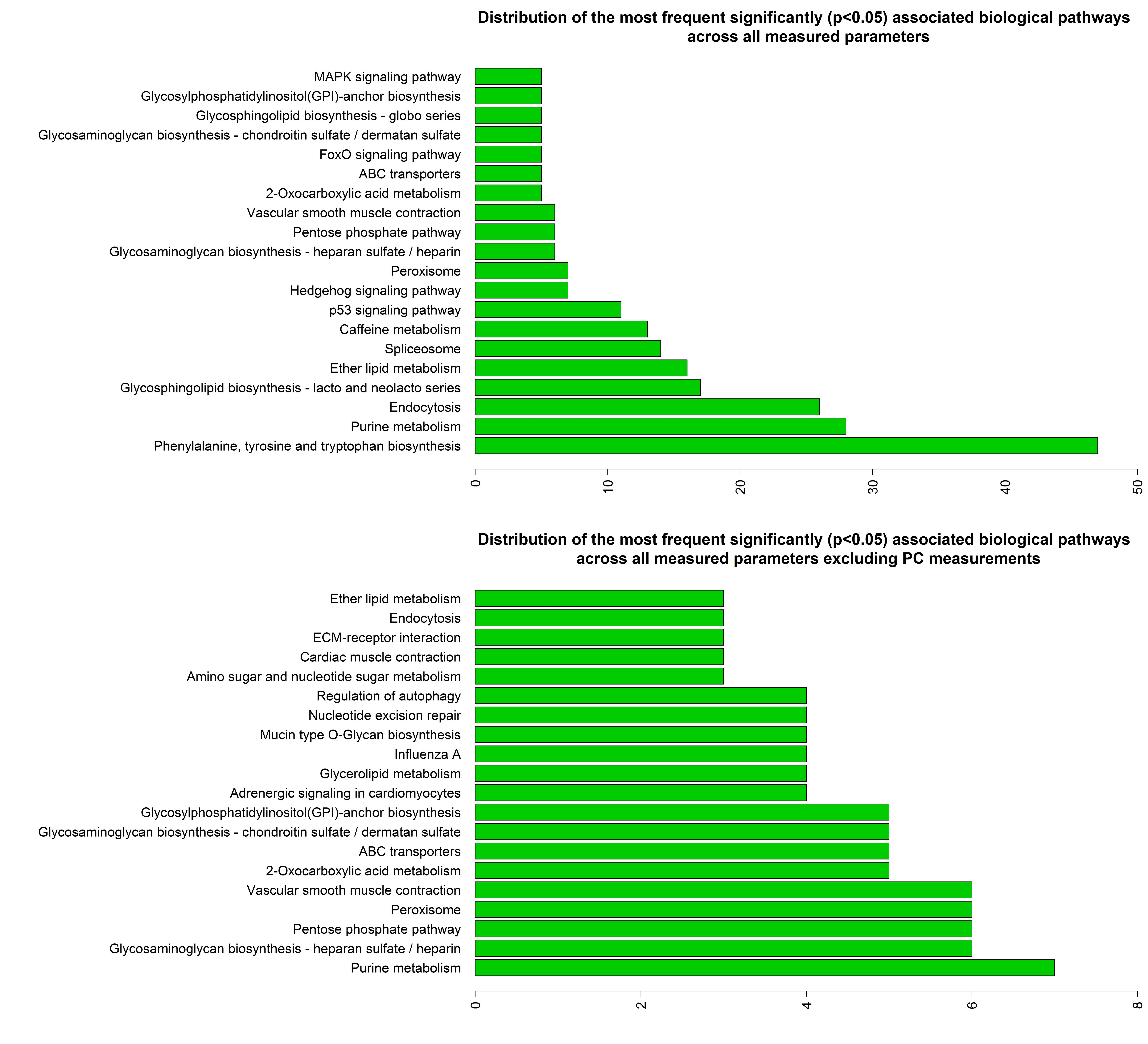
Distribution of the most frequent significantly (p<0.05) associated biological pathways across all measured parameters
MAPK signaling pathway
Glycosylphosphatidylinositol(GPI)-anchor biosynthesis
Glycosphingolipid biosynthesis - globo series
Glycosaminoglycan biosynthesis - chondroitin sulfate / dermatan sulfate
FoxO signaling pathway
ABC transporters
2-Oxocarboxylic acid metabolism
Vascular smooth muscle contraction
Pentose phosphate pathway
Glycosaminoglycan biosynthesis - heparan sulfate / heparin
Peroxisome
Hedgehog signaling pathway
p53 signaling pathway
Caffeine metabolism
Spliceosome
Ether lipid metabolism
Glycosphingolipid biosynthesis - lacto and neolacto series
Endocytosis
Purine metabolism
Phenylalanine, tyrosine and tryptophan biosynthesis
0
10
20
30
40
50
Distribution of the most frequent significantly (p<0.05) associated biological pathways across all measured parameters excluding PC measurements
Ether lipid metabolism
Endocytosis
ECM-receptor interaction
Cardiac muscle contraction
Amino sugar and nucleotide sugar metabolism
Regulation of autophagy
Nucleotide excision repair
Mucin type O-Glycan biosynthesis
Influenza A
Glycerolipid metabolism
Adrenergic signaling in cardiomyocytes
Glycosylphosphatidylinositol(GPI)-anchor biosynthesis
Glycosaminoglycan biosynthesis - chondroitin sulfate / dermatan sulfate
ABC transporters
2-Oxocarboxylic acid metabolism
Vascular smooth muscle contraction
Peroxisome
Pentose phosphate pathway
Glycosaminoglycan biosynthesis - heparan sulfate / heparin
Purine metabolism
0
2
4
6
8
