# Supplementary material for: Genome-wide association study and biological pathway analysis of the Eimeria maxima response in broilers
Source: Genet Sel Evol. 2015 Nov 25;47:91. doi: 10.1186/s12711-015-0170-0 (PMC4659166; doi:10.1186/s12711-015-0170-0)
Supplement: Supplementary file 12 — 10.1186/s12711-015-0170-0 Title: Illustration of the genomic region between 28.95 and 29.11 Mb on GGA5. Description: The genomic region between 28.95 and 29.11 Mb contains three SNPs that were significantly associated with body weight gain (BWG) and also contains several chicken spliced mRNA and EST, which may indicate the presence of a non-annotated chicken gene. Illustration was obtained from the UCSC Genome Browser (ICGSC Gallus_gallus-4.0/galGal4). [file 12711_2015_170_MOESM12_ESM.pdf]

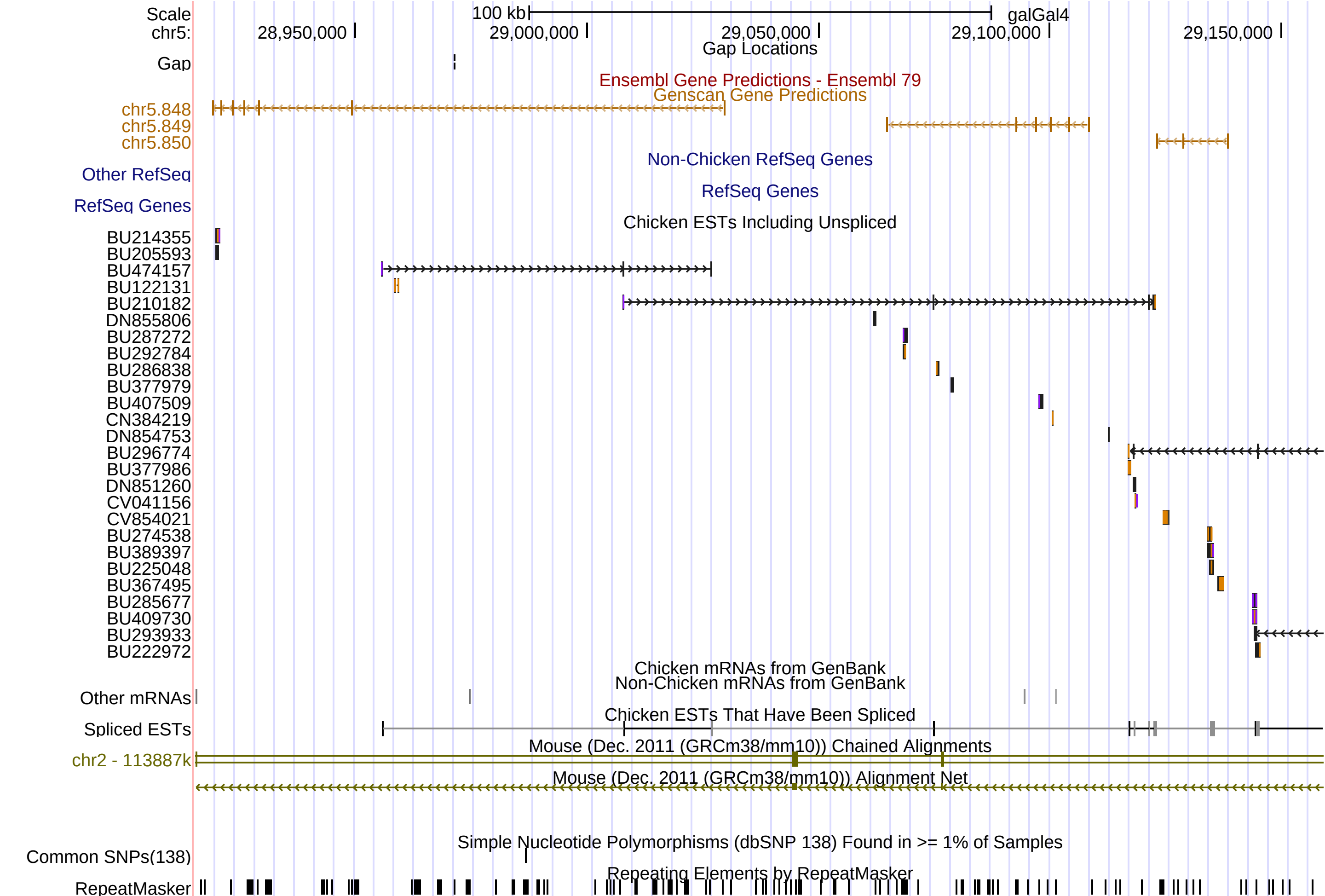
Scale
100 kb
galGal4
chr5:
28,950,000
29,000,000
29,050,000
29,100,000
29,150,000
Gap Locations
Gap
Ensembl Gene Predictions - Ensembl 79
Genscan Gene Predictions
chr5.848
chr5.849
chr5.850
Non-Chicken RefSeq Genes
Other RefSeq
RefSeq Genes
RefSeq Genes
Chicken ESTs Including Unspliced
BU214355
BU205593
BU474157
BU122131
BU210182
DN855806
BU287272
BU292784
BU286838
BU377979
BU407509
CN384219
DN854753
BU296774
BU377986
DN851260
CV041156
CV854021
BU274538
BU389397
BU225048
BU367495
BU285677
BU409730
BU293933
BU222972
Chicken mRNAs from GenBank
Non-Chicken mRNAs from GenBank
Other mRNAs
Chicken ESTs That Have Been Spliced
Spliced ESTs
Mouse (Dec. 2011 (GRCm38/mm10)) Chained Alignments
chr2 - 113887k
Mouse (Dec. 2011 (GRCm38/mm10)) Alignment Net
Simple Nucleotide Polymorphisms (dbSNP 138) Found in >= 1% of Samples
Common SNPs(138)
Repeating Elements by RepeatMasker
RepeatMasker
